# Anthony Ng: the kids are alright

**DOI:** 10.1192/bjb.2021.27

**Published:** 2021-08

**Authors:** Claire McKenna

**Figure:**
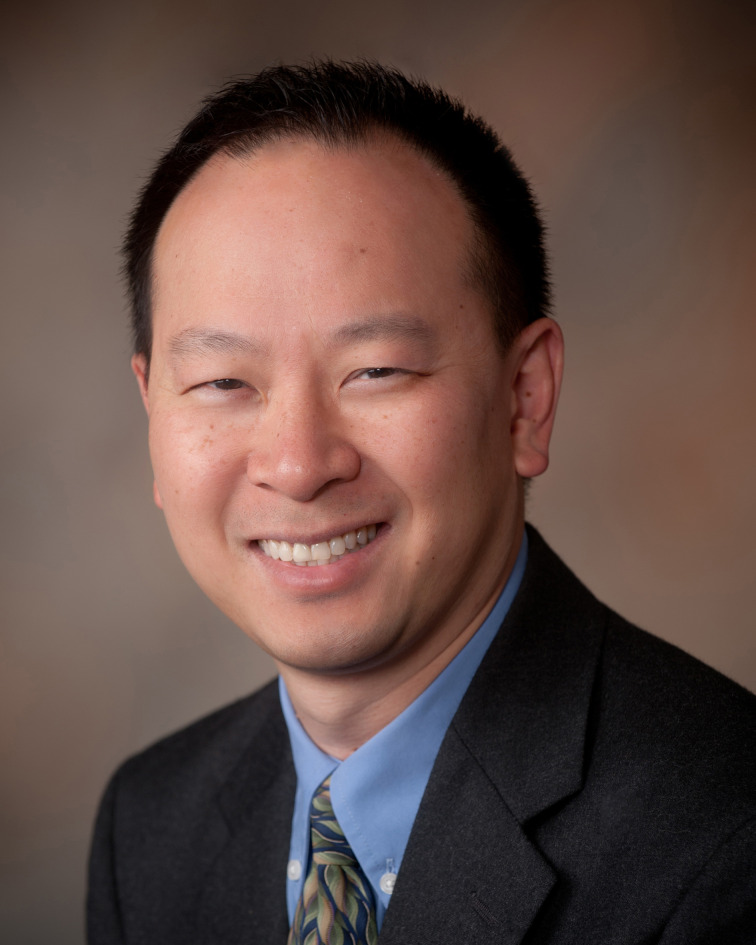

Dr Anthony ‘Tony’ Ng is a general psychiatrist and medical director of community services at Northern Light Acadia Hospital in Bangor, Maine, USA. He has a long interest in disaster emergency psychiatry and climate psychiatry and in 2019 took up a year-long role as the first chair of the American Psychiatric Association's (APA's) Caucus on Climate Change and Mental Health. He is the former chair of the APA's Committee on Psychiatric Dimensions of Disaster, with which he is still involved. In addition, he is a member of the Climate Psychiatry Alliance, an independent organisation (with members in the USA and UK), which works to lobby government and raise awareness of the links between climate change and mental health. Dr Ng is also a proud member of the Royal College of Psychiatrists.

Dr Ng has aided the psychological response to victims of disasters around the world and led a disaster response coalition, the New York City chapter of National Voluntary Organizations Active in Disaster (NYCVOAD), between 2001 and 2003. His work has involved him in atrocities such as the 9/11 terrorist attack in New York in 2001 and the Sandy Hook school shooting in Connecticut in 2012, as well as natural disasters such as Hurricane Katrina in 2005, among many others.

This interview took place via Zoom in September 2020 and has been edited for length and clarity.

**How did you come to be interested in the area of disaster relief and, more recently, climate psychiatry?**

Well, it began with an interest in trauma, because I realised so many of my patients in community mental health had some sort of trauma-related issues. I then became interested in humanitarian assistance for refugees and so forth. It was the 1990s and I think Bosnia, Croatia and Cambodia were pretty up there in the news. And I was mostly focused on the psychiatric piece, PTSD and so forth. As I'm doing this stuff, I realise that you can't really appreciate it until you can look beyond just the trauma – you need to look at how these things happened in the first place. And that got me interested in things such as socioeconomic factors, political strife and also climate issues. Climate became a big issue for me about 5 years ago, but it was brewing before that because I responded to [Hurricane] Katrina and I saw the aftermath of it all. When you start looking at it, it is climate change that leads to some of the severe weather that we've seen.

In the past few years, the APA started to take a lot more interest in the relationship between climate change and natural disasters and that led to the connection I have with the Climate Psychiatry Alliance. We wanted to explore what climate change is doing to our patients and what are the long-term effects it could have on psychiatric well-being in terms of population-based models, as well as the individual-based model.

**How have people in the USA been affected by disasters, both natural and man-made?**

Almost every year we're seeing increasing preparation for some sort of weather issue, whether its tornadoes or hurricanes or wildfires. Ice storms can also be a big problem in the winter, which take out a fair bit of the country if they happen.

What has been challenging is that disasters based on terrorist attacks are the media-driven events; they don't happen often but they have high emotional content. And I think that's what draws a lot of the attention. But every year, every US state is struggling with some sort of weather-related event – they've kind of gotten used to it. But what people are not seeing is how often these hurricanes are coming, how intense they're coming and also why the risk of these events is going up. More people are migrating to the coasts for whatever reason. In essence, it puts them more at risk for trauma from natural disasters, which may be stemming from climate change. And so it's kind of a ripple effect. But it's a slow pain. People don't appreciate the impact as much.

**I wanted to hear a little bit more about your past work in disaster relief. Was there anything that stands out as a particular learning experience for you?**

I honestly think the best learning experience was the work that I did for 3 years in New York City after 9/11. Now, granted, it wasn't climate related, but what it really educated me on was the strong interface between disasters and mental health, along with the human service side. You really can't appreciate disaster mental health until you really develop a good understanding of human service response to disasters.

As a psychiatrist you think about the PTSD, depression, anxiety and all those things. Certainly, you can work with those. But when you look at it more deeply – people are worrying about food, shelter, job stability, legal issues – those become bigger issues for them. So, it is not as much about the diagnostic arena, but how do people in distress behave? However, the focus in psychiatry has always been on that small diagnostic corner and the research money goes into that area.

The majority of people don't go on to develop PTSD, but they certainly have a lot of distress behaviours that can affect their overall well-being. For example, after a disaster, how do I find my loved ones? How do I get information about what's going on? How do I seek care? I just lost my job, so who will assist me with resources? How we intervene in these areas early on has an impact much later.

One of the pieces of feedback we often hear from people is that it's not the disaster that created the stress, it's the disaster *after* the disaster.

**The Climate Psychiatry Alliance website alludes to the impact of climate change on ‘the theoretical foundations and research priorities of psychiatry’. Can you elaborate a little bit on how you think climate change might have an on impact those areas?**

With climate change, we have not had the investment to really look specifically at what some of the markers or correlations are that might impact on people's mental health. So, I think this is one of the charges of the Climate Psychiatry Alliance – to increase people's awareness of the need to fund research.

One of the biggest achievements it [the CPA] was able to do is to create a Climate Change Caucus within the American Psychiatric Association, which sits under the Disaster Committee. They were able to advocate for it to become a standing entity. The other thing about the entity is that it falls under the Council of Research within the APA, so that means it also gives them a little bit more face time in terms of encouraging a focus on further research. Certainly, we need a better research base for climate change and mental health. And people are slowly doing it.

But the challenge, of course, is that the return isn't as fast as some of the other psychiatric research. Climate change in general is a creeping disaster. It's a death by a thousand cuts kind of thing if you don't do anything about it. And the healing process also takes a long time. So, though your results may not be earth shattering within a year or two, certainly over time they could add to the overall resources we have to deal with climate change and better understand the resources that we need to help people with it.

**So, you were the first chair of the Caucus on Climate Change?**

I was very honoured to be asked to chair the first year to really kind of get it going a little bit. It's only about 3 years old now. And the other nice thing about it is that, because of the work of the Caucus, the disaster course which the APA gives to its members (basically teaching about basic principles of disaster psychiatry) has added a section on climate change impact and disaster psychiatry.

**So, you can point to some tangible gains. It seems that you see the functions of the Caucus as raising the profile of climate change psychiatry and influencing research priorities. Are there any other areas you think are important for the Caucus or for the Climate Psychiatry Alliance?**

We also worked on areas such as lobbying. One of our members, for example, she lives in DC [Washington DC] and she has significant knowledge about advocacy to legislators on climate change issues. She has become a resource for local politicians because Congress is right there.

One nice thing about the Climate Psychiatry Alliance that's different from the Climate Caucus is that the Climate Caucus sits with the APA. So, you kind of have to do things within what the APA can do within its lane. But the Climate Psychiatry Alliance allows many more opportunities to engage without representing official positions.

**Do you think it's important for us as psychiatrists to take an interest in climate change?**

I believe it is important to appreciate its impact. I think to be a better psychiatrist you do have to have some appreciation of climate change, especially perhaps climate change where you're working.

It may be a big task to try to understand climate change globally. But I think for every psychiatrist, I think there's some impact within our areas. So, if you work in a rural area, you should know what climate change is doing to your rural population, for example if there's a change to the crop cycle, if there's a change to the drought season. If you're in the city as a psychiatrist, how does it affect your patients there? Does it increase the risk of allergies by affecting air quality for example?

In a city environment, you may have more people in the population who may appreciate climate change. Outside that, they may not have the same feeling, but your farmer may have just lost a job because of severe drought in your area. It's not that you're blatantly up front saying ‘I want to talk about climate change’, but you're saying ‘I could see in the back of my mind how climate change may be impacting your life and what stresses you go through’.

**One of the problems with addressing climate change is that it's sometimes hard to see the wood for the trees, so your suggestion that we focus on the small area that we can make a difference in sounds helpful. Do you have any advice for psychiatrists generally about what they could do on an individual level and at a system level to combat the climate crisis?**

It's helpful if you can identify what local resources are available for you where you're living. Let's say, for example, using my wife's island (she is originally from the Isle of Man), you need to know what the Isle of Man initiatives are. One of the areas they're looking at is a reduction in the use of peat for heating.

The use of peat creates a significant amount of carbon emissions. How is that impacting your local community? And these are very easy things to look up. I think then you can appreciate the impact because, for example, yeah you can reduce peat use, but how does it affect your patient who is trying to get heat? Because peat may be the cheapest thing right now for them. You can help to at least frame the discussion with your patients.

And also, I think certainly it's helpful to encourage more like-minded advocacy. For example, could we have a similar Caucus on Climate Change in the UK for psychiatrists?

**Would that be a good idea, do you think?**

I think we should work closely. I think, first of all, such a group can encourage the Royal College [of Psychiatrists] to put more on the climate change agenda that will trickle down to what individual psychiatrists do, but that also can ripple down to the research that you and I were just talking about earlier. There's a lot on for the leadership and because there are so many things going on, they may not see this as a big priority for them.

And maybe the *local* psychiatric association can get together and say, ‘OK, what is the impact of climate change in Northern Ireland?’. I know that by me doing something, I feel like I'm contributing to a solution rather than sitting there on the sideline.

**Do you think climate change has a disproportionate effect on people with mental disorders?**

I believe so for many reasons. For people who have mental illness in general, they are often already disadvantaged socioeconomically. I can move away to an area that's nicer so that it can be less impactful on me in terms of climate change. People with mental illness are often stuck. Their coping mechanisms are challenged because of mental illness. Whatever anxiety and stress they may deal with from other ripple effects, whether it is just higher heating bills and other related stresses, they don't have as many resources to help them navigate through those challenges. We might have the option to say, ‘I do my part because I buy organic meat and can buy socially responsible groceries’. But if you live in a neighbourhood where organic options are not available you don't have that choice.

**One of the things we're seeing is a spike in climate-related anxiety, particularly among children. Do you have any thoughts about how we can talk to kids about climate change without making them really anxious about it?**

As much as we adults try to think about this, kids are much more concerned. They're much more savvy about what's going on than we are. And I think they are a very good barometer for us. In terms of talking to kids, I think it's a conversation that needs to happen. And it's OK if you don't have all the answers. I don't think kids expect you to have all the answers. I think the piece I would really instil in them is to understand what science is and what facts are. If I can ask my kids to do some critical thinking, I'm setting them up so that in 10, 15 years time, they would ask these same questions in the same way that we would like them to.

**That brings us to ‘alternative facts’! A lot of us in the UK have watched with dismay as Trump reneged on the Paris climate agreement, et cetera. Are you more pessimistic now than you were about the chances of the USA addressing climate change in a meaningful way?**

I think, first of all, it is very hard for some folks to appreciate what climate change is. They live in, let's say, a small village. If you talk about corals, they never see corals. It doesn't affect them. It doesn't mean anything to them. I think one thing we fail at in terms of having those discussions about climate change is how climate change affects you *there,* where you are, versus climate change in general. And when people can't relate to it, it becomes harder for them to empathise and say, ‘I have to do something about it’.

But this is not just us. I think everywhere is struggling with that, too. One interesting thing about the model of government we have here in the US is that there's a lot of state government versus federal government. So, yes, while the federal government pulled out of the Paris Agreement, there are a lot of governors who basically said, ‘We're not – that's going to be our benchmark’. For example, in the state of Maine, the governor has created a task force on climate change. She wants to reduce emissions, wants to improve climate change and she wants to aim for the goals of the Paris Agreement. So, I think that's the little bit of hope that we have.

**Which leads me neatly on to my final question. There is sometimes a lot of pessimism around climate change for people who do believe in it, that actually we're not moving fast enough to make meaningful change. How hopeful in general are you about whether humanity can turn away from climate disaster?**

Maybe I'm too optimistic, but I think as the human race, we will move toward a better place. Are we going to have bumps along the way? I can guarantee you we will but I do think folks are increasingly seeing some of these critical issues. I think for a lot of reasons, economics will drive it. I mean, you can look at history. Climate change, yes, it's not easy, but I can still remember when I was growing up in the 1970s seeing Environmental Protection Agency commercials of people dumping toxic waste and trash in rivers and now we have made significant progress in those areas. So, I think it's going to happen slowly.

**You clearly *are* an optimist.**

Yes and no. To some degree, I am pragmatic. I think what really helped me personally is having a 10-year-old son. I tell him that sometimes he's the best teacher for me. He reminds me of certain things, because you know, we rationalise all kinds of reasons for why we do certain things, but the kids bring it down to core values. So, I think this is what we need to do.

As much as people feel frustrated that we can't make big changes right now, 5 years ago I didn't know about Greta [Thunberg], I didn't know about those kids. Now, I hear about kids taking legal action, asking for injunctions on certain initiatives that they feel may be damaging to the climate. That's a big deal. And as I said, over time, these kids vote. Over time these kids become leaders in whatever field they're going to and will have an impact. To change the culture, you need a couple of generations before you can move it through.

Climate change, it's unfortunate, but it's going to be a marathon, not a sprint, so we've got to be prepared for it.

